# Stability Test of PACAP in Eye Drops

**DOI:** 10.1007/s12031-020-01532-9

**Published:** 2020-04-22

**Authors:** Anita K. Kovacs, Tamas Atlasz, Dora Werling, Edina Szabo, Dora Reglodi, Gabor K. Toth

**Affiliations:** 1 Department of Medical Chemistry, Faculty of Medicine, University of Szeged, Dom Sq 8, Szeged, H-6720 Hungary; 2 Department of Anatomy, MTA-PTE PACAP Research Group, Medical School, University of Pecs, Szigeti str 12, Pecs, H-7624 Hungary; 3 Department of Sportbiology, University of Pecs, Ifjusag str 6, Pecs, H-7624 Hungary; 4 Department of Ophthalmology, Medical School, University of Pecs, Rakoczi str 2, Pecs, H-7623 Hungary

**Keywords:** PACAP, Eye drops, Stability, Degradation

## Abstract

PACAP is a neuropeptide with widespread distribution and diverse biological functions. It has strong cytoprotective effects mediated mainly through specific PAC1 receptors. Experimental data show protective effects of PACAP in the retina and cornea in several pathological conditions. Although intravitreal injections are a common practice in some ocular diseases, delivery of therapeutic agents in the form of eye drops would be more convenient and would lead to fewer side effects. We have previously shown that PACAP, in the form of eye drops, is able to pass through the ocular barriers and can exert retinoprotective effects. As eye drops represent a promising form of administration of PACAP in ocular diseases, it is important to investigate the stability of PACAP in solutions used in eye drops. In this study, the stability of PACAP1-27 and PACAP1-38 in eye drops was measured in four common media and a commercially available artificial tear solution at both room temperature and +4 °C. Mass spectrometry results show that the highest stability was gained with PACAP1-38 in water and 0.9% saline solution at +4 °C, representing 80–90% drug persistence after 2 weeks. PACAP1-38 in the artificial tear showed very fast degradation at room temperature, but was stable at +4 °C. In summary, PACAP1-38 has higher stability than PACAP1-27, with highest stability at +4 °C in water solution, but both peptides in each medium can be stored for relatively longer periods without significant degradation. These data can provide reference for future therapeutic use of PACAP in eye drops.

## Introduction

The neuropeptide pituitary adenylate cyclase activating polypeptide (PACAP) exists in two active forms, PACAP1-38 and PACAP1-27, both of which are well-established neuro- and general cytoprotective peptides, with 38 and 27 amino acid residues, respectively (Reglodi et al. 2017, 2018a, b; Shioda and Nakamachi 2015). PACAP1-27 is the form with the shorter C-terminal, representing about 10% of the naturally occurring peptide (Miyata et al. 1990; Vaudry et al. 2009). Both PACAP forms occur in most organs, with the highest expression levels in the nervous system, endocrine glands and testis, but several peripheral organs also have measurable levels of PACAP (Fulop et al. 2019; Horvath et al. 2019; Reglodi et al. 2018c; Vaudry et al. 2009). PACAP and its receptors are also found in ocular tissues, including the lacrimal gland, conjunctiva, inner eye muscles and different layers of the eye. It has been found in all three layers of the eyecup: the fibrous, vascular and nervous layers (Atlasz et al. 2016; Seki et al. 2000a, b). PACAP exerts several different effects in the eye. It affects tear secretion (Nakamachi et al. 2016), influences muscle responses of the iris (Yamaji et al. 2005), increases blood flow in the eye (Dorner et al. 1998) and regulates pigment epithelial cell functions (Fabian et al. 2012, 2019; Maugeri et al. 2019a). Most importantly, as a general protective peptide found not only in the central nervous system but several peripheral organs as well (Laszlo et al. 2019; Liu et al. 2019; Polanco and Pennuto 2018; Reglodi et al. 2018d, e; Shioda et al. 2019; Szegeczki et al. 2019), PACAP has been shown to exert diverse retinoprotective effects in models of toxic, ischemic, inflammatory and traumatic retinal injuries (Atlasz et al. 2016, 2019; Cheng et al. 2018; Endo et al. 2011; Gabriel et al. 2019; Kvarik et al. 2016; Seki et al. 2008; Szabadfi et al. 2016; Vaczy et al. 2016; Ye et al. 2019a, b). Several retinal cell types can be protected by PACAP, including ganglion cells, bipolar neurons, amacrine and pigment epithelial cells (Atlasz et al. 2008; Fabian et al. 2019; Maugeri et al. 2019a; Szabadfi et al. 2012).

PACAP has protective effects not only in the retina, but also in the cornea, where PACAP and its receptors are present in the cornea (Maugeri et al. 2018, 2019b, c; Wang et al. 1995). A few studies have investigated the local effects of PACAP on the cornea. A study in rabbits found that PACAP1-27 eye drops promoted the growth of neuronal processes in the cornea and accelerated recovery of corneal sensitivity (Fukiage et al. 2007). Although it focused only on neuronal recovery, the study drew attention to the possibility that PACAP, in the form of eye drops, could enhance corneal recovery. Indeed, the enhancement of corneal regeneration by topical administration of PACAP was subsequently confirmed in two independent studies (Ma et al. 2015; Nakamachi et al. 2016). PACAP has also demonstrated protective effects on corneal endothelial cells, indicating an important trophic function of the peptide in the cornea (Maugeri et al. 2018, 2019b, c). In in vivo studies, PACAP was given in the form of eye drops in order to exert local effects on the cornea. In contrast, most studies showing retinoprotective effects of the peptide have utilized intravitreal administration. Intravitreal injections, despite their wide clinical use, have the distinct disadvantage of being invasive (Atlasz et al. 2016; Shioda and Nakamachi 2015). Recently, in a model of ischemic retinopathy, we provided evidence that both PACAP forms, given as eye drops, are able to pass through the ocular barriers and reach the retina, where they can exert retinoprotective effects (Werling et al. 2016, 2017). This shows that PACAP treatment as eye drops is a promising therapeutic approach not only in corneal diseases, but also in retinal pathologies. Therefore, it is important to investigate the stability of PACAP in different solutions used in ophthalmic practice. Since the ocular application of PACAP is a potential therapeutic approach in several diseases, including dry eye syndrome (Shioda et al. 2019), the aim of the present study was to analyze the stability of PACAP1-27 and PACAP1-38 in the most commonly used eye drop solvents.

## Materials and Methods

### Materials

PACAP1-27 and PACAP1-38 were synthesized in our laboratory on a CEM Liberty microwave peptide synthesizer (Matthews, NC, USA) and were dissolved in the following sterile vehicles: (i) 0.9% saline solution, (ii) benzalkonium chloride solution for ophthalmic use (SOCB), (iii) thimerosal solution for ophthalmic use and (iv) water for injection, obtained from the Faculty Central Pharmacy, Faculty of Medicine, University of Szeged. A commercially available artificial tear solution (Systane Ultra^®^, Alcon, Switzerland) was also used in the experiment.

Analytical reversed-phase high-performance liquid chromatography (RP-HPLC) was performed on an Agilent 1200 Series separation system with diode array and multiple wavelength detector (Waldbronn, Germany), with a Luna C18(2) 100 Å column (10 μm, 250 × 4.6 mm; Phenomenex, Torrance, CA, USA). The chromatography was carried out at room temperature (RT), with a flow rate maintained at 1.2 mL min^−1^ at a wavelength of 220 nm [mobile phase solvent A: 0.1% TFA in Milli-Q water; solvent B: 0.1% TFA in acetonitrile (AcN)] using gradient elution. Mass spectrometry (MS) data were collected on a Waters SQ Detector (Milford, MA, USA) with an API mass spectrometer in positive ion mode.

### Peptide Synthesis and Purification

For the experiment, the synthesized peptides at the University of Szeged (Szeged, Hungary) were as follows: PACAP1-27: H-His-Ser-Asp-Gly-Ile-Phe-Thr-Asp-Ser-Tyr-Ser-Arg-Tyr-Arg-Lys-Gln-Met-Ala-Val-Lys-Lys-Tyr-Leu-Ala-Ala-Val-Leu-NH_2_; PACAP1-38: H-His-Ser-Asp-Gly-Ile-Phe-Thr-Asp-Ser-Tyr-Ser-Arg-Tyr-Arg-Lys-Gln-Met-Ala-Val-Lys-Lys-Tyr-Leu-Ala-Ala-Val-Leu-Gly-Lys-Arg-Tyr-Lys-Gln-Arg-Val-Lys-Asn-Lys-NH_2_. The sequences were synthesized by a solid-phase technique utilizing Fmoc (fluorenylmethyloxycarbonyl) chemistry. The peptide chains were elongated on a Rink amide MBHA resin (1.1 mmol/g), and the syntheses were carried out using a CEM Liberty microwave peptide synthesizer. The side-chain protecting groups were as follows: Fmoc-His(Trt) (Trt: trityl), Fmoc-Ser(tBu) (tBu: tert-butyl), Fmoc-Asp(tBu), Fmoc-Thr(tBu), Fmoc-Tyr(tBu), Fmoc-Arg(Pbf) (Pbf: 2,2,4,6,7-pentamethyldihydrobenzofuran-5-sulfonyl), Fmoc-Lys(Boc) (Boc: tert-butyloxycarbonyl). Coupling was performed with HBTU. The completed peptide resins were treated with TFA/water/TIS (93:5:2, *v*/v) at RT for 2.5 h. The reagents were removed, and the resulting free peptides were solubilized in 10% aqueous acetic acid, filtered and lyophilized. Next, 120–150 mg of crude peptides was dissolved in 1.5 mL 5% m/m acetic acid, and then filtered using a 0.45 μm nylon filter. Gradient elution was used, 20–40% eluent B for 50 min at a flow rate of 3 mL min^−1^, with detection at 220 nm. Pure fractions were collected and lyophilized to give a white material, with weight of 55–63 mg.

### Stability Testing

The stability of the peptides was examined with LC-MS in four media commonly used in ophthalmology: (i) 0.9% saline solution, (ii) benzalkonium chloride solution for ophthalmic use (SOCB), (iii) thimerosal solution for ophthalmic use and (iv) water for injection. First, 0.5 mg peptide was dissolved in 0.5 mL solvent; after dissolution, the resulting liquids were halved. One half of the solvent was cooled to and maintained at +4 °C; the other half was kept at RT. After 3, 6, 8, 11 and 14 days, 40 μL of the given solutions was examined. The stability of PACAP1-38, which showed higher stability in every condition, was also tested in a commercially available artificial tear solution as medium [ingredients: polyethylene glycol 400, propylene glycol, hydroxypropyl guar, sorbitol, aminomethyl propanol, potassium chloride, sodium chloride, 0.001% Polyquad^®^ (polidronium chloride)], following the same protocol.

## Results

**Fig. 1:**
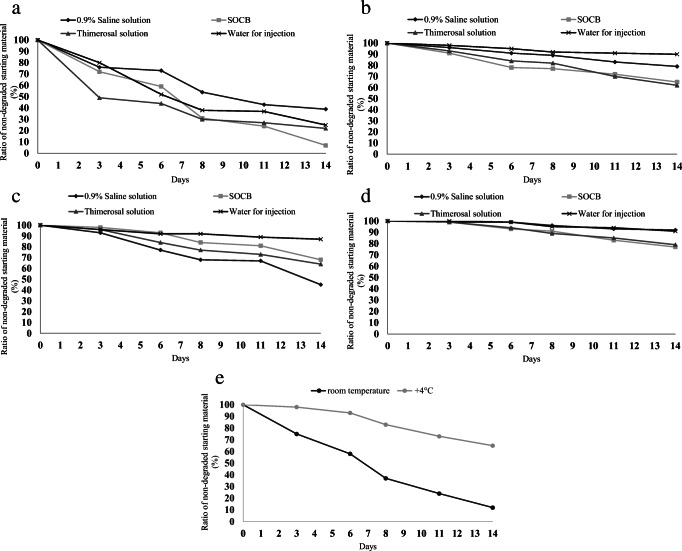
Degradation of PACAP1-27 and PACAP1-38 at room temperature (RT) and at +4 °C in four media (0.9% saline solution, SOCB, thimerosal solution, water for injection) (**a**–**d**). We found that all four solutions demonstrated significantly higher stability at +4 °C than at RT, and the rate of degradation was higher in the SOCB and thimerosal solutions than in saline or water vehicles. PACAP1-38 was also more stable than PACAP1-27 in the four media. Degradation of PACAP1-38 in Systane^®^ Ultra at RT and +4 °C (**e**). Higher stability was found at +4 °C, similar to the other examined media

**Fig. 2:**
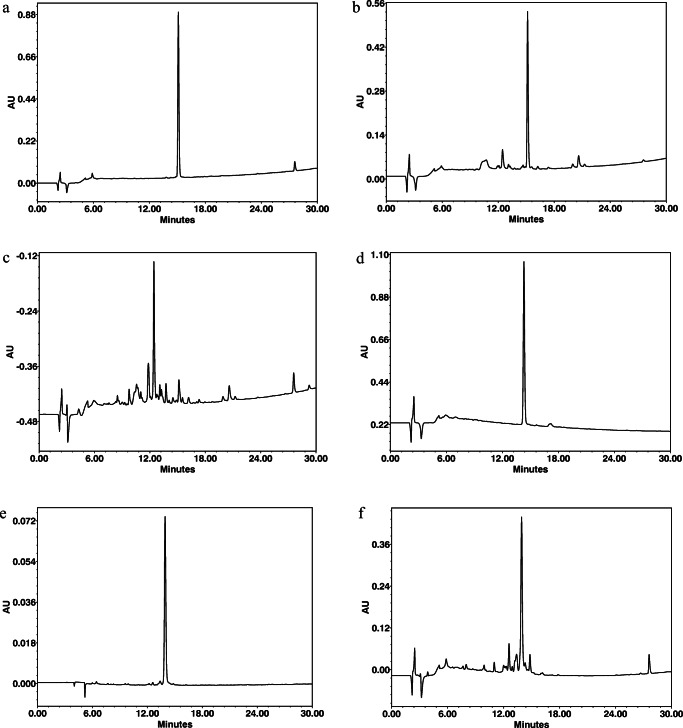
PACAP1-27 initial state t_R_ = 15.14 min (**a**). The most stable PACAP1-27 after day 14 medium: water for injection, temperature: +4 °C (**b**). The most degraded PACAP1-27 after day 14 medium: SOCB, temperature: room temperature (**c**). PACAP1-38 initial state t_R_ = 13.94 min (**d**). The most stable PACAP1-38 after day 14 medium: water for injection, temperature: +4 °C (**e**). The most degraded PACAP1-38 after day 14 medium: SOCB, temperature: room temperature (**f**). Conditions: 0–100% B in 30 min, 220 nm, 1.2 mL/min, eluent A: 0.1% TFA/H_2_O, eluent B: 80% AcN/0.1% TFA/H_2_O

Table 1 and Figs. 1 and 2 show the stability results for PACAP1-27 in the four media at the two experimental temperatures (RT and +4 °C) over a 2-week period. The results show that at +4 °C, all four solutions have significantly higher stability than the solutions at RT, and the rate of degradation is higher in the SOCB and thimerosal solution than in the other two vehicles (0.9% saline and water). While more than 90% of PACAP1-27 was still intact at +4 °C after 14 days, only 25% remained un-degraded at RT. In contrast, PACAP1-27 was almost completely degraded in benzalkonium chloride solution at RT, while 65% remained intact at the colder temperature.

**Table 1:** Stability of PACAP1-27 in different media and conditions over a period of 2 weeks. The numbers in the cells indicate the percentage of the starting material that was not decomposed on the given day

PACAP1-27	0.9% Saline solution		Benzalkonium chloride solution for ophthalmic use (SOCB)		Thimerosal solution for ophthalmic use		Water for injection	
	RT	+4 °C	RT	+4 °C	RT	+4 °C	RT	+4 °C
Day 3	76	96	72	91	49	93	80	98
Day 6	73	91	59	78	44	84	52	95
Day 8	54	89	31	77	30	82	38	92
Day 11	43	83	24	72	27	70	37	91
Day 14	39	79	7	65	22	62	25	90

*RT* room temperature

PACAP1-38 solutions proved to be more stable than PACAP1-27 in the same four media under the same thermal conditions (Table 2, Figs. 3 and 4). At +4 °C, all four solutions retained more than 90% of PACAP1-38 un-degraded, and even after 2 weeks, more than 90% of the original peptide was measured in saline and water solutions, and more than 75% in the other two solutions.

**Table 2:** Stability of PACAP1-38 in different media and conditions over a period of 2 weeks. The numbers in the cells indicate the percentage of the starting material that was not decomposed on the given day

PACAP1-38	0.9% Saline solution		Benzalkonium chloride solution for ophthalmic use (SOCB)		Thimerosal solution for ophthalmic use		Water for injection	
	RT	+4 °C	RT	+4 °C	RT	+4 °C	RT	+4 °C
Day 3	93	99	98	99	96	99	96	100
Day 6	77	99	93	93	84	94	92	99
Day 8	68	96	84	91	77	89	92	95
Day 11	67	93	81	83	73	85	89	94
Day 14	45	92	68	77	64	79	87	91

*RT* room temperature

PACAP1-38 stability was also measured in a commercially available artificial tear solution (Systane^®^ Ultra) at the two experimental temperatures over a 2-week period (Table 3 and Fig. 5). The results showed that the lower temperature gave higher stability, similar to the other examined solutions, but values were worse than in the other solvents during the second week.

**Table 3:** Stability of PACAP1-38 in a commercially available artificial tear solution (Systane^®^ Ultra). Numbers in the cells indicate the percentage of the starting material that was not decomposed on the given day

PACAP1-38	Artificial tears (Systane^®^ Ultra)	
	RT	+4 °C
Day 3	75	98
Day 6	58	93
Day 8	37	83
Day 11	24	73
Day 14	12	65

*RT* room temperature

LC-MS measurements showed that a lower temperature (+4 °C) resulted in higher stability for both peptides in all media, but PACAP1-38 had higher stability than PACAP1-27 in all media at both experimental temperatures. Both PACAP1-27 and PACAP1-38 solutions with 0.9% saline solution and water for injection were more stable at both temperatures throughout the 14-day period. We can conclude that the stability of PACAP1-38 and PACAP1-27 was highly medium-dependent. We examined the more stable PACAP1-38 in a commercially available artificial tear solution as medium and found that the stability was lower than in any of the other media. Our findings were confirmed with RP-HPLC profiles of both the initial state and the least and most degraded PACAP1-27 and PACAP1-38 (Figs. 6–11). PACAP1-38 proved to be completely stable in water for injection at +4 °C over a period of 2 weeks (Fig. 10).

## Discussion

In the present study we showed the time course of degradation of PACAP1-27 and PACAP1-38 in different solutions at room temperature and at +4 °C. The results show that PACAP1-38 has significantly higher stability than PACAP1-27 at both RT and +4 °C in each medium, with the longest stability in 0.9% saline solution and water for injection.

Naturally occurring or exogenously injected PACAP1-38 and 1-27 are degraded by several peptidases in the blood (Bourgault et al. 2009). Dipeptidyl peptidase IV (DPPIV) cleaves PACAP1-38 to the PAC1 receptor antagonist PACAP3-38 and 5–38 fragments, while PACAP1-27 is more resistant to DPPIV but is readily cleaved by neutral endopeptidase, similar to the structurally homologous VIP. Other enzymes also take part in further cleavage, such as carboxypeptidase and endopeptidase and prohormone convertase (Bourgault et al. 2009; Gourlet et al. 1997).

The therapeutic value of PACAP and/or its derivatives has emerged in light of its strong neuroprotective and general cytoprotective properties as well as potent vasodilatory and several other biological effects (Cline et al. 2019; Jozsa et al. 2019; Parsons and May 2019; Reglodi et al. 2018a; Shioda et al. 2019; Van et al. 2019; Vaudry et al. 2009). PACAP has shown in vivo protective effects in animal models of cerebral ischemia, Parkinson’s and Alzheimer’s disease, Huntington chorea, traumatic brain and spinal cord injury, and different retinal pathologies (Reglodi et al. 2017, 2018b). PACAP passes through the blood–brain barrier (Banks 2016), and therefore, systemic administration can affect the nervous system and lead to neuroprotective effects. Several other routes of administration have been proven to provide protective effects of PACAP in the nervous system and peripheral organs, such as intracerebral, intrathecal, intracerebroventricular, intravitreal and systemic treatments, as well as intravenous, intraperitoneal and subcutaneous administration. Other options include emerging therapeutic approaches such as intranasal and eye drop treatments (Cabezas-Llobet et al. 2018; Meredith et al. 2015; Reglodi et al. 2018a). As far as protection in the eye is concerned, the intravitreal approach is the first choice for treatment in animal models of ocular diseases (Atlasz et al. 2016; Kiss et al. 2006; Reglodi et al. 2018a). This approach has led to the demonstration of the retinoprotective effects of PACAP in models of retinal hypoperfusion (Atlasz et al. 2007), traumatic optic nerve injury (Seki et al. 2008), kainate- and glutamate-induced excitotoxicity (Atlasz et al. 2009; Seki et al. 2006), UV light-induced lesion (Atlasz et al. 2011), lipopolysaccharide-induced inflammation (Vaczy et al. 2018), oxygen-induced retinopathy of prematurity (Kvarik et al. 2016), diabetic retinopathy (D’Amico et al. 2017; Szabadfi et al. 2016) and high intraocular pressure-induced retinopathy (Seki et al. 2011).

Although intravitreal treatments are commonly used in ophthalmological practice, it is an invasive method, with potential side effects and patient discomfort. PACAP, in the form of eye drops, has been shown to lead to extension of neuronal processes from amputated nerve trunks in the cornea following laser-assisted in situ keratomileusis and to accelerate recovery of corneal sensitivity after the surgery (Fukiage et al. 2007). Corneal application of PACAP1-27 eye drops or of a PACAP-derived peptide, with higher stability and PAC1-specific potency than PACAP, also led to enhancement of corneal wound healing in mice (Ma et al. 2015). PACAP treatment in the form of eye drops is also able to increase tear secretion and cAMP and pPKA levels, in addition to the suppression of corneal keratinization and dose-dependent corneal wound healing in mice and rats (Farkas et al. 2010; Nakamachi et al. 2016). We recently showed that both PACAP1-27 and PACAP1-38 given in the form of eye drops could readily cross the ocular surfaces and could reach the retina in a concentration high enough to exert retinoprotective effects in a model of retinal ischemia (Werling et al. 2016, 2017). These results offer a potential novel therapeutic approach to treating retinal diseases. The use of PACAP in eye drops, therefore, would be beneficial not only in corneal diseases, but also in retinal pathologies. The emerging potential of PACAP in the form of eye drops led us to investigate the degradation process of PACAP1-27 and PACAP1-38 in the most commonly used solvents at two different temperatures, room temperature and +4 °C, which are important from both an experimental and clinical perspective. The present results provide a future reference for PACAP solutions to be used in the treatment of ocular disease.
